# MiRNA-802 suppresses proliferation and migration of epithelial ovarian cancer cells by targeting YWHAZ

**DOI:** 10.1186/s13048-019-0576-3

**Published:** 2019-10-22

**Authors:** Bo Yang, Li Sun, Lei Liang

**Affiliations:** Department of Obstetrics and Gynecology, Bethune International Peace Hospital, 398 Zhongshan West Road, Shijiazhuang, 050000 Hebei Province China

**Keywords:** Epithelial ovarian cancer, miR-802, YWHAZ, Proliferation, Metastasis

## Abstract

**Background:**

The imbalance of expression of microRNA-802 may have a significant place in tumor progression. However, the bio-function of epithelial ovarian cancer cells remains unclear. Therefore, we setup this study to explore the pathogenesis of epithelial ovarian cancer based on microRNA-802.

**Methods:**

RT-qPCR analysis was used to measure the expression level of microRNA802 and YWHAZ in epithelial ovarian cancer. CCK-8, colony formation, flow cytometry and transwell assay were used to detect the effects of microRNA-802 on cell proliferation, apoptosis, invasion and migration. Target gene prediction and screening, luciferase reporting experiments were applied to validate the downstream target genes of microRNA-802. The effects of microRNA-802 on the expression of YWHAZ and its biological effects were measured by Western blotting and RT-qPCR.

**Results:**

Compared with normal cell lines and tissues, the expression level of microRNA-802 was obviously down-regulated in cancer related cell lines and tissues. Overexpression of microRNA-802 could obviously inhibit the invasion and proliferation and induce apoptosis. In addition, YWHAZ was the binding target protein of miR-802 for epithelial ovarian cancer cells. YWHAZ was obviously up-regulated in human epithelial ovarian cancer cells, and YWHAZ was negatively correlated with the expression of miR-802. YWHAZ can partly eliminate the inhibitory effect caused by overexpression of miR-802 on growth and metastasis of epithelial ovarian cancer cells.

**Conclusion:**

miR-802 can regulate the occurrence and development of epithelial ovarian cancer by targeting YWHAZ.

## Background

The mortality rate of ovarian malignant tumors ranks first in gynecological malignant tumors [1, 2]. Epithelial ovarian cancer is a popular ovarian cancer, about 85 to 90% of ovarian cancer belong to this cancer type [3]. People have been studying ovarian cancer for more than 150 years. Unfortunately, the mortality rate of ovarian cancer has not decreased. The anatomical location of the ovary is hidden, and the early symptoms are not obvious [4, 5]. In recent years, although the surgical treatment and chemotherapy of epithelial ovarian cancer (OC) have been improving constantly [6, 7]. Therefore, how to find and control the related factors of proliferation, invasion and metastasis, and then improve the survival rate of patients with epithelial ovarian cancer has become a research hotspot.

Many scholars have done a lot of research on epithelial ovarian cancer, and current research focuses on gene regulation levels, including DNA methylation, group egg protein modification, and non-coding RNA, among which non-coding RNA has received unprecedented attention [8, 9]. Studies have showed that miRNAs have a key place in almost all important life activities [10, 11]. At present, it has been found that it is related to many diseases, among which the identification of miRNAs in tumors and the exploration of their functions have become the hotspot and frontier of life science research [12]. MicroRNAs (miRNAs) are a class of small (19 to 25 nt), non-coding, highly stable RNAs that regulate mRNA and protein expression [13]. Moreover, they should specify that several studies have indicated that miRNAs have been involved in regulating various biological processes, such as cellular differentiation, proliferation, angiogenesis, metabolism and cancer development [14, 15]. Many kinds of miRNAs have been proved to be abnormally appeared in carcinoma of ovary. MiRNAs are involved in biological processes related to the development of ovarian cancer [16]. The study has also found that miRNAs are associated with staging, grading, and histological subtypes of ovarian cancer, suggesting that miRNAs can be screened for ovarian cancer as a class of biomarkers [17, 18]. Some studies have showed that miRNA-802 has obvious differences in the expression levels of various tumor cells and surrounding healthy tissues [19, 20]. However, current research on miRNA-802 in ovarian cancer is rarely reported.

In recent years, research have found that miRNAs make a difference in biological through downstream target genes [21]. YWHAZ belongs to the 14–3-3 gene family. Studies have found that YWHAZ have a vital function in tumor migration, inhibited apoptosis and regulated signal transduction [22, 23]. It is abnormally expressed in many malignant tumor cells [24]. The YWHAZ mechanism of action on the development of ovarian carcinoma is not clear. Based on the above studies, it was hypothesized that miRNA-802 may regulate the development of ovarian cancer through YWHAZ expression. This study main purpose was to attest the mechanism of miRNA-802 regulation of ovarian cancer, to verify the role and relationship of YWHAZ in miRNA-802-regulated ovarian carcinoma, and to demonstrate a theoretical basis for finding new drug targets.

## Methods

### Research object

Matched detection of epithelial ovarian cancer and adjacent normal tissues was performed in 35 patients who underwent surgical resection in Bethune International Peace Hospital. This study was approved by the ethics committee of Bethune International Peace Hospital and was conducted in accordance with the Helsinki Declaration. All patients signed written informed consent. None of the patients received radiotherapy or chemotherapy. The clinicopathological features of these patients were summarized in Table 1.

**Table 1:** Clinicopathological features of epithelial ovarian cancer patients

Features	*N*. of patients (*n* = 35)
Age	
< 55 years	16
≥ 55 years	19
Histological type	
Serous	11
Mucinous	15
Others	9
Histological grade	
G1	18
G2	7
G3	10
Residual tumor upon surgery	
< 1 cm	12
≥ 1 cm	23
FIGO stage	
I/II	10
III/IV	25

### Cell culture

HOSEpiC cells (normal human ovarian surface epithelial) and Epithelial ovarian cancer cells (OVCAR3, SKOV3, A2780 and CAOV3) were obtained from the Shanghai Cell Bank of Chinese Academy of Sciences.

### miRNA and plasmid transfection

Both miR-802 mimic (miR-802) and miR-NC (negative control) were obtained from GenePharma Company (Shanghai, China). The sequence used was as follows: miR-802 sense, 5′-CAGUAACAAAGAUUCAUCCUUGU-3′; miR-NC 5′-CAGUACUUUUGUGUAGUACAA-3′. YWHAZ was over expressed by synthesis of the pcDNA3.1-YWHAZ overexpression plasmid (GenePharma), the empty vector severed as a negative control. Stable cell lines were then infected with miR-802/NC (50 nM) or pcDNA3.1-YWHAZ plasmid/pcDNA3.1 (2 μg) with Lipofectamine 2000 reagent (Invitrogen) and OptiMEM (Invitrogen). The cells were harvested 36 h post-transfection for subsequent experiments.

### Quantitative real-time PCR (qRT-PCR)

Total RNA from cells was obtained with TRIzol reagent (Guyu, Shanghai, China). Once the reverse transcription reaction was done, qRT-PCR was started using a ViiATM 7 real-time PCR system (Jinuo, Shanghai, China). GAPDH and U6 were treated as inner benchmarks. Quantitative real-time PCR (qRT-PCR) specific experimental methods were performed with reference to the literature [25]. The primer sequences were as follows:

1. miR-802-forward 5′-CGTTGTGTAGCTTATCAGACTG-3′;
2. miR-802-reverse 5′-AATGGTTGTTCTCCACACTCTC-3′;
3. U6-forward 5′-CTCGCTTCGGCAGCACA-3′;
4. U6-reverse 5′-AACGCTTCAGGAATTTGCGT-3′;
5. YWHAZ-forward 5′-AGGAGCCCGTAGGTCATCTT-3′;
6. YWHAZ-reverse 5′-TGCTTGTGAAGCATTGGGGA-3′;
7. GAPDH-forward 5′-AAGGTGAAGGTCGGAGTCAAC-3′;
8. GAPDH-reverse 5′-GGGGTCATTGATGGCAACAATA-3′

### Cell proliferation assay

Cell Counting Kit-8 was used for analysis the cell proliferation (CCK8; Dojindo, Tokyo, Japan). The transfected cell proliferation was measured every one day. The absorbance values were finally measured at 450 nm with an ELx 800 microplate reader (Bio-Tek, USA).

### Colony formation test

After 2 weeks, cells were washed, fixed by methanol and then stained with crystal violet (1%). Finally, the amount of colonies was calculated under inverted microscope. (Olympus, Japan).

### Migration and invasion assay

Transwell chamber with 8 μm pore size (Corning Incorporated, Corning, NY, USA) was used for cell migration and invasion detection. The upper basement membrane of the Transwell chamber was pre-coated with 20 μg Matrigel Matrigel and cultured overnight in a 24-well plate. Cell suspension was placed in the upper chamber and culture medium in the lower chamber. After 12 h of culture, it was washed with PBS for 3 times. Then it was fixed with 90% of the formaldehyde, and stained in the crystal violet solution. In the cell migration experiment, the upper chamber of the Transwell chamber was free of matrigel coating, and the rest of the operation was the same as the invasion experiment.

### Apoptosis analysis

After 36 h of cell transfection, epithelial ovarian cancer cells were collected. Then they were incubated with 5 μl of propidium iodide (PI) and 5 μl of FITC Annexin V. The apoptotic rate was determined by flow cytometry (FACS Calibur, USA). Specific experimental methods were carried out in reference to the literature [26].

### Dual luciferase reporter gene assay

The potential target protein or genes of miR-802 protein were identified by bioinformatics methods using the online software program starBase v2.0. Wild-type miR-802 (miR-802 WT) and YWHAZ 3′-UTR WT (3′-UTR YWHAZ) reporter plasmid or mutant reporter plasmids (miR-802 MT and YWHAZ 3′-UTR MT) with pmirGLO fluorescein enzyme vector (Promega, WI) were constructed, and cells were transfected with luciferase reporter plasmids(a wild type or mutant plasmid). Luciferase activity was measured by double luciferase assay system kit (Promega).

### Western blot analysis

The transfected cells were collected, total protein was extracted. Then the protein was electrophoresised and transferred to the membrane of PVDF. After adding anti- YWHAZ (ab13589, abcam), anti-GAPDH (ab16321, abcam), the 1:5000 labeled secondary anti-rabbit antibody was added for 1 h, and the specific experimental method of Western blot analysis was performed by reference to the literature [27].

### Statistical method

The monitoring data were analyzed by SPSS19.0 statistical software. The results of data analysis were showed as mean ± standard deviation (mean ± SD). Multigroup data analysis was based on one-way ANOVA. LSD test was used for subsequent analysis. *P* < 0.05 meant the difference was significant.

## Results

### Expression of miR-802 is down regulated in epithelial ovarian cancer cell lines and tissues

**Fig. 1:**
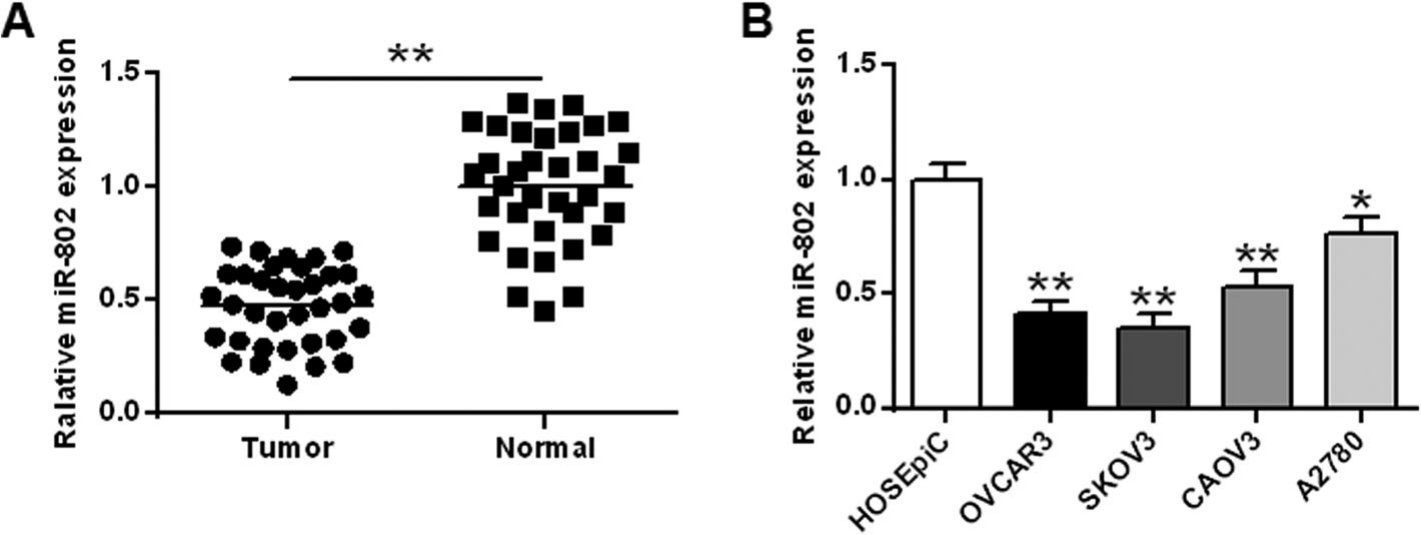
Levels of miR-802 expression. **a** Expression levels of miR-802 in epithelial ovarian cancer tissues and adjacent normal tissues. **b** miR-802 expression levels in epithelial ovarian cancer cell lines and normal human ovarian surface epithelial cells.* *P* < 0.05, ** *P* < 0.01

First, the expression level of miR-802 gene in epithelial ovarian cancer tissues was analyzed. As shown in Fig. 1a, the expression yield of miR-802 gene in epithelial ovarian cancer tissues was significantly lower than that in normal tissues (*P* < 0.01). In addition, miR-802 expression was significantly reduced in 4 human epithelial ovarian cancer tumor cells (OVCAR3, SKOV3, A2780 and CAOV3) compared with that in the normal control cell line HOSEpiC, which were consistent with tissue assay results (*P* < 0.01) (Fig. 1b). Since the expression yield of mir-802 gene in OVCAR3 and SKOV3 cell lines were relatively low, the OVCAR3 and SKOV3 cell lines were selected as candidate cell models for further studies in this study.

### Overexpression of miR-802 suppressed migration, proliferation, invasion and induces apoptosis in epithelial ovarian cancer

**Fig. 2:**
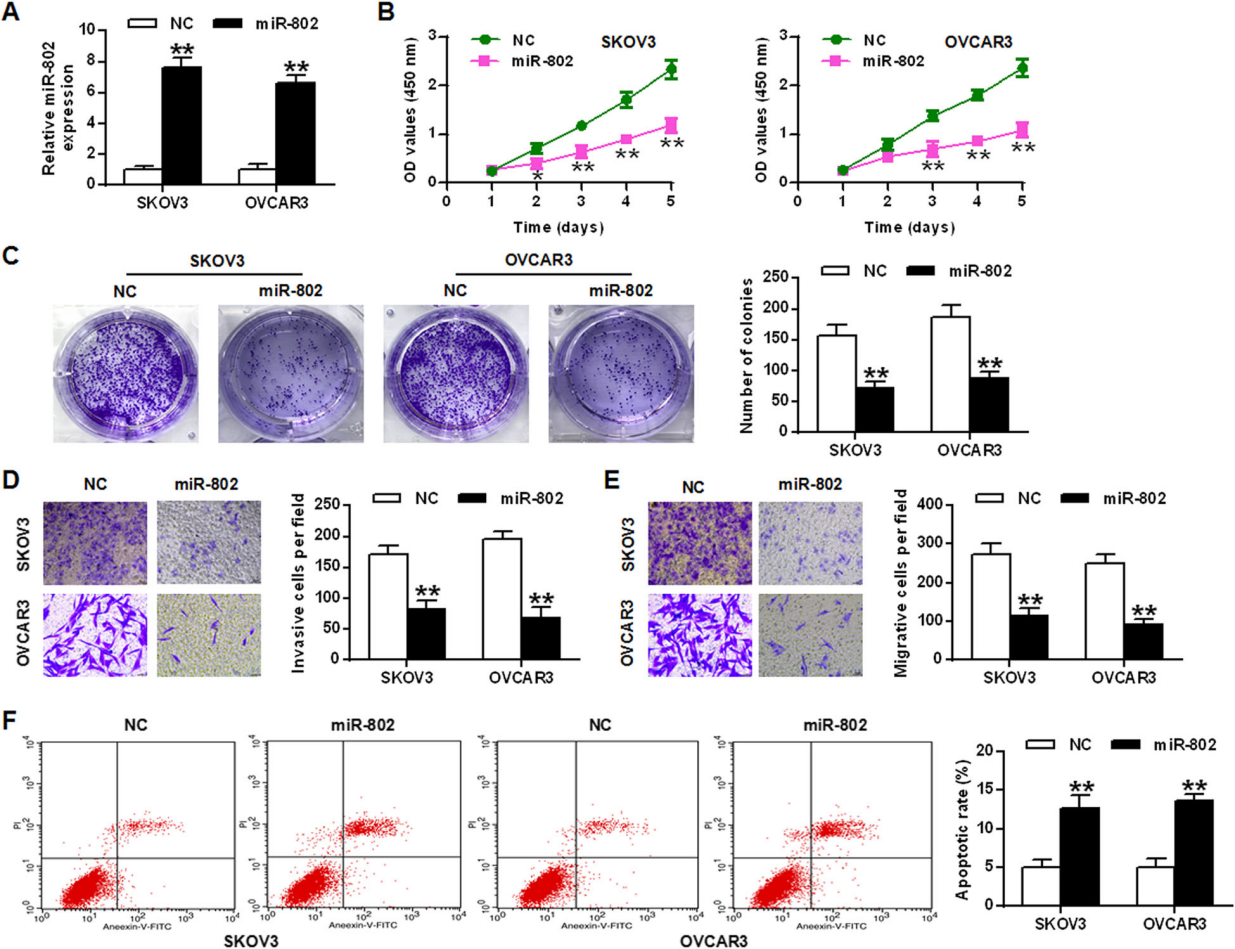
Biological effects of miR-802 on epithelial ovarian cancer cells. **a** Expression levels of miR-802 in SKOV3 and OVCAR3 cells. **b** Cell growth rate in SKOV3 and OVCAR3 cells. **c** Colony forming ability of SKOV3 and OVCAR3 cells. Cell invasion (**d**) and migration ability (**e**) were measured in SKOV3 and OVCAR3 cells. **f** Apoptosis rate of SKOV3 and OVCAR3 cells.* *P* < 0.05, ** *P* < 0.01

The data were indicated in Fig. 2a. The expression yield of miR-802 gene was obviously increased after LV-UCA1 transfection, indicating successful transfection (*P* < 0.01).

The CCK8 assay indicated that the proliferation of OVCAR3 and SKOV3 cells was obviously inhibited in the miR-802 gene overexpression group with a contrast to that of the NC (negative control) group (*P* < 0.05, *P* < 0.01) (Fig. 2b). Colony formation results showed that in the miR-802 overexpression group, the number of colonies of SKOV3 and OVCAR3 cells was obviously decreased with a contrast to that in the NC (negative control) group (*P* < 0.01) (Fig. 2c). Transwell assay results indicated that the miR-802 overexpression group obviously suppressed the invasion and migration of OVCAR3 and SKOV3 cells with a contrast to that of the NC (negative control) group (*P* < 0.01) (Fig. 2d and e). Flow cytometry results indicated that with a contrast to NC group, the apoptosis rate of SKOV3 and OVCAR3 cells was obviously enhanced in the miR-802 overexpression group (*P* < 0.01) (Fig. 2f). In summary, the above data showed that miR-802 exerted a tumor suppressor effect and inhibited the metastasis and growth of epithelial ovarian cancer cells.

### YWHAZ was a direct target gene of miR-802 in epithelial ovarian cancer cells

**Fig. 3:**
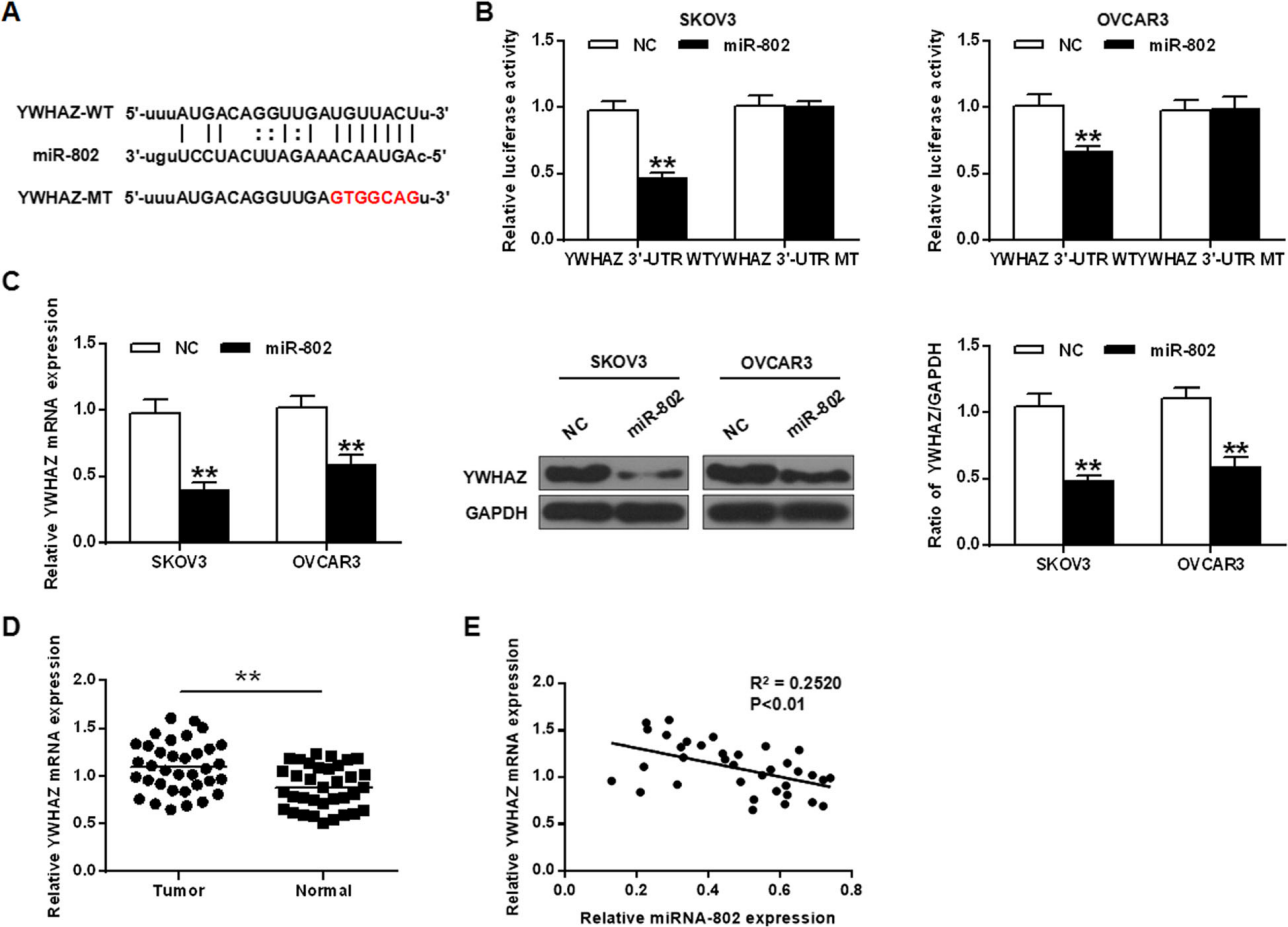
YWHAZ was a direct target of miR-802 in epithelial ovarian cancer cells. **a** Bioinformatics analysis revealed predicted binding sites between miR-802 and YWHAZ. **b** Relative luciferase activity in cells co-transfected with miR-802 mimic or NC and pLUC-YWHAZ 3′-UTR wild type (WT) or pLUC-YWHAZ 3′-UTR mutant (MT) active. **c** The levels of YWHAZ in SKOV3 and OVCAR3 cells. **d** Expression levels of YWHAZ in epithelial ovarian cancer tissues and adjacent normal tissues. **e** Spearman correlation analysis of the correlation between miR-802 expression and YWHAZ mRNA expression.** *P* < 0.01

YWHAZ was predicted and identified as a potential target protein for miR-802 by bioinformatics (Fig. 3a). Luciferase reporter assays were performed to validate the predicted results using the WT-miR-802 or mutant (mut)-miR-802 luciferase reporter plasmid. As a result in Fig. 3b, in SKOV3 and OVCAR3 cells, ectopic expression of YWHAZ significantly inhibited luciferase activity of WT-miR-802, which had no significant effect on luciferase activity of mut-miR-802. Next, the expression pattern of miR-802 and YWHAZ in epithelial ovarian cancer cells was analyzed. As indicated in Fig. 3c, in OVCAR3 and SKOV3 cells, YWHAZ mRNA transcriptional and protein expression yields were significantly reduced in the miR-802 overexpressing group with a contrast to NC group (*P* < 0.01). Compared with normal tissues, YWHAZ expression was obviously up regulated in epithelial ovarian cancer tissues (*P* < 0.01) (Fig. 3d). In addition, a direct negative correlation between miR-802 expression and YWHAZ levels was found in epithelial ovarian cancer tissues (Fig. 3e). These results suggested that miR-802 protein may have its biological function in epithelial ovarian cancer through YWHAZ.

### Restoration of YWHAZ expression abrogated the impact of miR-802 on epithelial ovarian cancer cell proliferation and metastasis

**Fig. 4:**
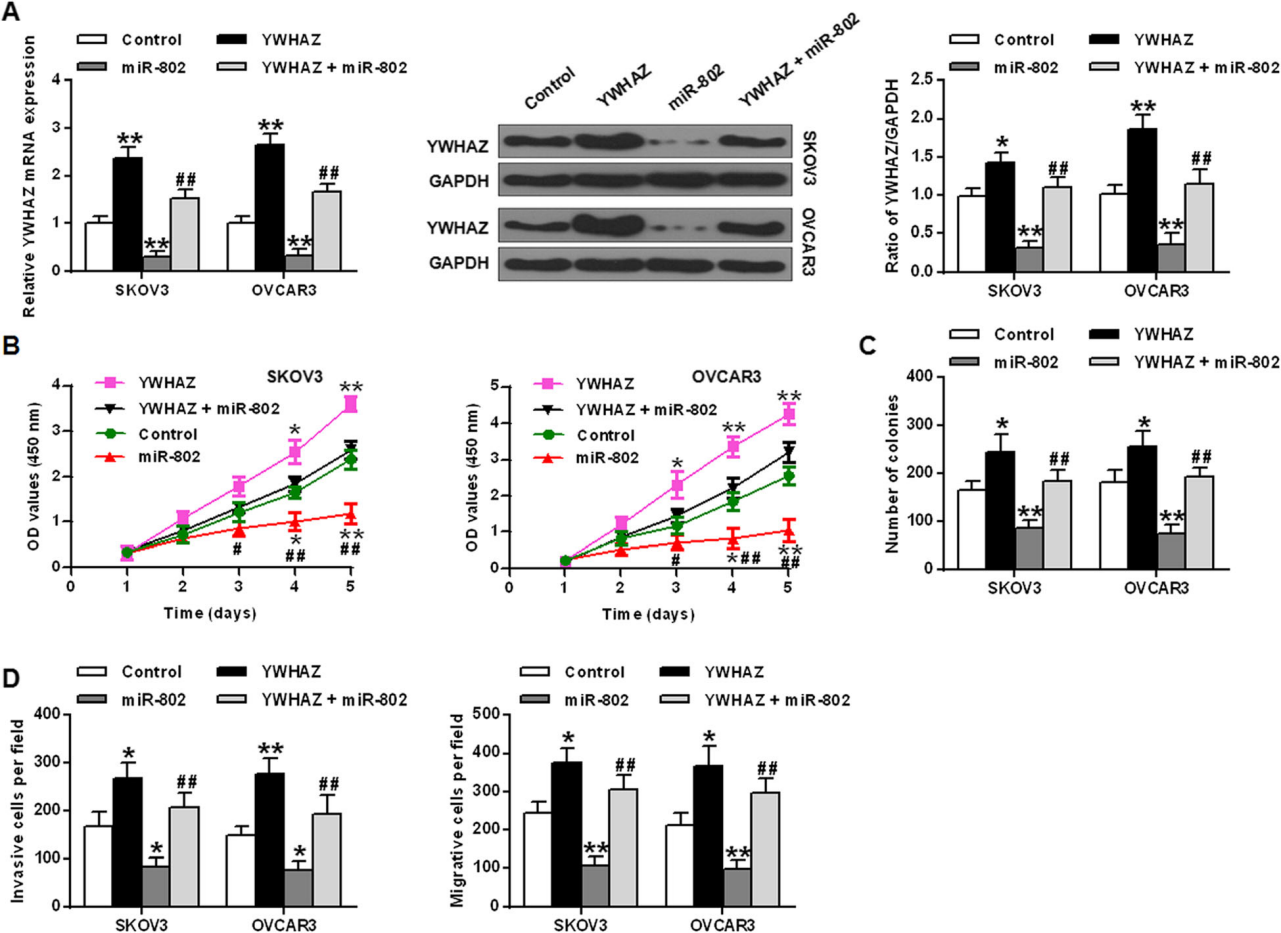
Overexpression of YWHAZ reversed the biological effects of miR-802 on epithelial ovarian cancer cells. **a** The levels of YWHAZ with miR-802 mimic and pcDNA3.1-YWHAZ. **b** Cell growth rate with miR-802 mimic and pcDNA3.1-YWHAZ. **c** Colony forming ability of SKOV3 and OVCAR3 cells co-transfected with miR-802 mimic and pcDNA3.1-YWHAZ. **d** miR-802 mimic and pcDNA3.1-YWHAZ co-transfected SKOV3 and OVCAR3 cells Invasion and migration ability with miR-802 mimic and pcDNA3.1-YWHAZ.* *P* < 0.05, ** *P* < 0.01 vs the control group; #*P* < 0.05, ## *P* < 0.01 vs the miR-802 group

The results were shown in Fig. 4a. In OVCAR3 and SKOV3cells, YWHAZ mRNA transcriptional and protein expression yields were obviously up regulated in the YWHAZ overexpression group with a contrast to that in the NC group (*P* < 0.05, *P* < 0.01). In the miR-802 overexpression group, YWHAZ mRNA transcriptional and protein expression yields were significantly reduced (*P* < 0.05, *P* < 0.01). Co-transfection of miR-802 and YWHAZ relieved the inhibited effect of miR-802 overexpression on YWHAZ expression levels (*P* < 0.01). CCK8 results and colony formation experiments indicated that in SKOV3 and OVCAR3 cells, YWHAZ overexpression obviously promoted cell growth rate and increased cell colony number with a contrast to NC group (*P* < 0.05, *P* < 0.01). MiR-802 overexpression significantly suppressed the cell growth rate and decreased the number of cell colonies (*P* < 0.05, *P* < 0.01). However, co-transfection and co-expression of miR-802 with YWHAZ reversed the inhibition effect of cell growth and colony formation by miR-802 overexpression (*P* < 0.05, *P* < 0.01) (Fig. 4b and c). In addition, in OVCAR3 and SKOV3 cells, the cell invasion and migration ability of the YWHAZ overexpression group was significantly increased with a contrast to that in the NC group (*P* < 0.05, *P* < 0.01), and the cell invasion and migration ability in the miR-802 gene overexpression group was significant decreased (*P* < 0.05, *P* < 0.01). However, co-transfection of miR-802 with YWHAZ abolished the inhibition of cell invasion and migration by miR-802 overexpression (*P* < 0.01) (Fig. 4d).

## Discussion

Ovarian cancer has the highest mortality rate among malignant tumors of female reproductive system [28]. Although the current surgical + combined chemotherapy treatment has become more mature, it often causes treatment failure due to chemotherapy resistance and recurrence and metastasis, and its five-year survival rate is still very low [29]. Therefore, it is very important to find new effective treatments. With the advancement of molecular biology, molecular targeted therapy has become a new treatment for malignant tumors [30]. Compared with traditional chemotherapy drugs, molecular targeted therapy has the characteristics of strong specificity, obvious curative effect, and less damage to normal tissue cells [31]. Ovarian cancer is a multi-gene, multi-step, multi-stage interaction development process [32]. Therefore, a better study of the molecular mechanism of ovarian cancer has important clinical value for the early diagnosis and effective treatment of ovarian cancer.

MicroRNAs (microRNAs) are a group of short-chain, non-coding single-stranded RNAs that directly target the untranslated regions (UTR) of mRNA and become a new post-transcriptional regulator [33]. More and more studies indicate that abnormal expression of miRNAs may be related to tumors [34]. Is it involved in tumorigenesis through regulatory genes and signaling pathways [35]. MiRNAs can regulate the development of ovarian cancer. Studies have found that in epithelial ovarian cancer, miR-15a, miR-16, and miR-31 expression are up-regulated, while miRNA-34, miRNA-100, and miR-199 are down-regulated [36, 37]. Therefore, it is speculated that the pathogenesis and mechanism of epithelial ovarian cancer is closely related to miRNA. MiR-802 is a research hotspot in recent years. Studies have shown that the expression level of miR-802 is abnormal in hepatocellular carcinoma, prostate cancer and bladder tumors [38]. This study found that the expression level of miR-802 gene in cancer cells and tissues was significantly reduced. The overexpression of miR-802 gene suppressed the invasion and proliferation in epithelial ovarian cancer cells. Based on the existing literature and our experimental results, we speculate that miR-802 may have a function of tumor suppressor gene in epithelial ovarian cancer. The decrease of miR-802 expression may trigger the activation of related anti-cancer mechanisms and inhibit the occurrence of epithelial ovarian cancer, which provides a theoretical basis for revealing the possible mechanism of epithelial ovarian carcinogenesis.

Numerous studies have indicated that miRNAs are related in tumorigenesis, regulation, and even prognosis through multiple signaling pathways [39]. For example, miR-802 regulates the proliferation of intestinal epithelial c2bbe1 cells by targeting Ang II signaling pathway [40]. YWHAZ is a highly conserved protein expressed in various tissues of human body. The YWHAZ gene affects cell proliferation under a number of pathological conditions [41]. Studies have found that miR-451 can inhibit hepatocellular carcinoma proliferation and metastasis by targeting YWHAZ [42]. It was screened YWHAZ as a target gene for miR-802 through a database. And miR-802 regulates its expression level by targeting the 3’UTR of the YWHAZ gene. The expression of miR-802 in cancer tissues was significantly increased, and a negative relationship between miR-802 expression and YWHAZ levels was observed. This study proved that the expression level of YWHAZ was obviously decreased in the miR-802 overexpression group, while the YWHAZ expression level was significantly enhanced in the miR-135a inhibitor group. MiR-802 silencing partially reversed the YWHAZ expression level in the miR-802 inhibitor group. There was also a direct positive relationship between miR-802 expression and YWHAZ levels. The expression yield of YWHAZ was obviously decreased in the miR-802 overexpression group, while co-transfection of miR-802 and YWHAZ reversed the effect of miR-802 overexpression on YWHAZ mRNA and protein expression levels. Moreover, YWHAZ overexpression promoted cell proliferation rate and increased the number of colonies and invasion and migration ability. MiR-802 overexpression inhibited cell proliferation rate and reduced the number of colonies and invasion and migration ability. However, co-transfection of miR-802 with YWHAZ reversed the inhibition of cell growth, invasion and migration by miR-802 overexpression. These indicated that miR-802 inhibited the progression of epithelial ovarian cancer by targeting YWHAZ. We speculate that the binding of miR-802 to YWHAZ gene transcription can inhibit the translation of YWHAZ protein and regulate the biological function of epithelial ovarian cancer cells. In the process of the occurrence and development of epithelial ovarian cancer, YWHAZ signaling pathway mediated by miR-802 may play a role, but the detailed regulatory mechanism needs further study.

## Conclusions

MiR-802 can suppress the growth, invasion and metastasis of epithelial ovarian cancer by binding to YWHAZ, suggesting that miR-802 may be a potential tumor suppressor gene of epithelial ovarian cancer. It would provide experimental basis for the clinical prognosis judgment and further targeted intervention therapy of this cancer.

## Data Availability

The datasets generated and/or analyzed during the current study are not publicly available due research design, but are available from the corresponding author on reasonable request.

## Abbreviations

HOSEpiC cells: Normal human ovarian surface epithelial
microRNAs: MicroRNAs
OC: Ovarian cancer
PI: Propidium iodide
UTR: Untranslated regions
